# Phosphorus Availability Affects the Photosynthesis and Antioxidant System of Contrasting Low-P-Tolerant Cotton Genotypes

**DOI:** 10.3390/antiox12020466

**Published:** 2023-02-12

**Authors:** Mirezhatijiang Kayoumu, Asif Iqbal, Noor Muhammad, Xiaotong Li, Leilei Li, Xiangru Wang, Huiping Gui, Qian Qi, Sijia Ruan, Ruishi Guo, Xiling Zhang, Meizhen Song, Qiang Dong

**Affiliations:** 1 Institute of Cotton Research of Chinese Academy of Agricultural Sciences/Zhengzhou Research Base, State Key Laboratory of Cotton Biology/School of Agricultural Sciences, Zhengzhou University, Anyang 455000, China; 2 Western Agricultural Research Center of Chinese Academy of Agricultural Sciences, Changji 831100, China; 3 Department of Agriculture, Hazara University, Mansehra 21120, Pakistan; 4 Rice Cultivation Laboratory, Rice Research Institute, Sichuan Agricultural University, Chengdu 611130, China

**Keywords:** cotton, P availability, photosynthesis, chlorophyll fluorescence, OJIP curves, antioxidant enzymes

## Abstract

Phosphorus (P) is an essential macronutrient, and an important component of plant metabolism. However, little is known about the effects of low P availability on P absorption, the photosynthetic electron transport chain, and the antioxidant system in cotton. This study used cotton genotypes (sensitive FJA and DLNTDH and tolerant BX014 and LuYuan343) with contrasting low-P tolerance in a hydroponic experiment under 15 µM, 50 µM, and 500 μM P concentrations. The results showed that low P availability reduced plant development and leaf area, shoot length, and dry weight in FJA and DLNADH, compared to BX014 and LuYuan343. The low P availability decreased the gas-exchange parameters such as the net photosynthetic rate, transpiration rate, and stomatal conductance, and increased the intercellular CO_2_ concentration. Chlorophyll a fluorescence demonstrated that the leaves’ absorption and trapped-energy flux were largely steady. In contrast, considerable gains in absorption and trapped-energy flux per reaction center resulted from decreases in the electron transport per reaction center under low-P conditions. In addition, low P availability reduced the activities of antioxidant enzymes and increased the content of malondialdehyde in the cotton genotypes, especially in FJA and DLNTDH. Moreover, low P availability reduced the activity of PEPC and generated a decline in the content of ATP and NADPH. Our research can provide a theoretical physiological basis for the growth and tolerance of cotton under low-P conditions.

## 1. Introduction

Phosphorus (P) is necessary for the plant metabolism of adenosine triphosphate (ATP), nicotinamide adenine dinucleotide phosphate hydrogen (NADPH), nucleic acids, and phospholipids, because they all have significant roles to play in plant growth, production, signal transduction, and photosynthesis [1,2]. Therefore, the need for input of P in agriculture is projected to increase, due to the requirement for increased food production and improved crop yields [3]. Estimates suggest that almost 30% of the world’s arable soils lack P and need to be fertilized with this element to increase yields [4]. Because of the limited availability of P in the soil and its limited transportation, plants are frequently vulnerable to P nutritional stress in both natural and agricultural ecosystems [1]. Moreover, P fertilizer has a much lower efficiency of usage. Typically, plants only absorb 10–25% of the P fertilizer applied, whereas the residual P fertilizer leads to the deterioration of soil ecology [5]. Understanding the physiological and metabolic mechanisms of plants under different P availability to improve the utilization of P fertilizers is the key to solving the above problems.

Photosynthesis is an essential process that generates energy to support plant growth. Components participating in photosynthetic machinery, such as photosynthetic pigments, photosystems, electron-transport systems, gas-exchange processes, and enzymes involved in carbon metabolism, are important for photosynthetic efficiency [6,7]. Photosynthesis commonly declines under P deficiency, since P is involved in several cellular processes, including energy conservation, metabolic regulation, and signal transduction [8]. P deficiency can reduce the stomatal opening. With less stomatal opening, less CO_2_ is captured and reduced to triose phosphate, significantly reducing the recycling of ATP and NADPH, thus inhibiting plants’ photosynthetic capacity and growth [9]. Under P deficiency, photosynthesis is generally reduced, due to feedback inhibition resulting from reduced leaf growth. These decreases in photosynthetic activity might increase photosynthetic P-use efficiency and may also decrease the leaf-mass-per-unit leaf area. [10]. Plant species, and even genotypes of certain species, differ in their ability to maintain their photosynthetic activity under P limitation. Significant interspecific and intraspecific variation exists for photosynthetic P-use efficiency under low P availability [11,12].

Photosynthetic pigments play a central role in absorbing light and transmitting energy [13]. A deficiency of P also decreases photosynthetic-pigment contents of the leaf, such as carotenoids and chlorophyll (Chl) [14]. A previous study showed that P deficiency decreases the activity of ribulose-1,5-bisphosphate carboxylase/oxygenase (Rubisco) in diatom (*Phaeodactylum tricornutum*) and cotton (*Gossypium hirsutum* L.) [15,16]. Most studies claim that a shortage of P results in a decrease in net CO_2_ assimilation, which, in most instances, results in a decrease in stomatal conductance [17]. Phosphoenolpyruvate carboxylase (PEPC) is not only a key enzyme of C4 photosynthesis; other PEPC isoforms are also involved in a variety of metabolic processes in both photosynthetic and non-photosynthetic tissues of all plants, e.g., replenishment of the tricarboxylic-acid-cycle intermediates, participation in pH maintenance and regulation of guard-cell movement [18]. The low P availability reduces photosynthetic capacity by affecting the activities of enzymes involved in the Calvin cycle and ATP and NADP(H) [19,20]. This also results in a decreased use of reduction, NADP(H), and decreased ferredoxin Fd_red_ as a result of the decrease in Calvin-cycle activity. A low P availability also limits the electron transport chain (ETC), which, combined with an increase in the acidification of lumen pH induced by the decrease in ATP production, leads to the buildup of decreased electron carriers, such as plastoquinone. They also play a negative role in controlling the expression of enzymes in the Calvin-cycle enzyme and components of photosynthesis and ETC [21]. 

The chlorophyll-fluorescence (ChlF) parameters can change, which reflects how light energy is absorbed, used, transmitted, and dissipated by photosystem I (PSI) and photosystem II (PSII) [22]. The multiple reduction stages of the electron transport chain are reflected in the ChlF OJIP transient [23]. Previous studies have shown that the latent P deficiency can be revealed by using ChlF, which causes a change in the OJIP transient to form around the I-step [8]. Recently, a correlation was identified between the plant’s bioactive P pool and the appearance of the I-phase OJIP transient [8].

Chloroplasts convert light energy to energy for chemical bonds. Light absorption by the Chl molecules of PSI and PSII triggers a sequence of redox reactions along the thylakoid membrane. These reactions result in the oxidation of water, reduction of NADP^+^ to NADPH, and formation of a proton gradient across the thylakoid membrane. Reactive oxygen species (ROS) can be interconverted by interaction with redox-active compounds of the chloroplast, and ROS are produced both via O_2_-mediated primary ROS-generation mechanisms and ROS-mediated interconversion reactions [24]. ROS are unfavorable for chloroplast functions, because ROS cause oxidative damage by reacting with biomolecules [25]. Similar to other abiotic stresses, P deficiency inevitably leads to an increase in the production of ROS, a by-product of photosynthesis. Through the feedback-inhibition mechanism of sugar accumulation, the utilization of the ETC is reduced [26]. Plants have developed various defensive and remedial measures against this photooxidative stress, as well as major modifications to chloroplast biochemistry, such as highly regulated enzyme and non-enzyme mechanisms, including antioxidant enzymes, such as superoxide dismutase (SOD), peroxidase (POD) and catalase (CAT), to eliminate ROS and maintain the balance between ROS production and elimination [27]. The activity of these antioxidant enzymes will decrease under low-P-availability conditions [26,28]. Malondialdehyde (MDA) is a biomarker of lipid peroxidation found in plants or isolated chloroplasts, reflecting photooxidative damage to the lipids in the chloroplast membrane. It was found that the content of MDA increased under low-P conditions [29].

Cotton is a vital crop for the global economy that produces natural fiber for the textile industry. The agronomic properties of cotton are dramatically influenced by the low P availability. Although research on photosynthesis has been conducted in various plants [30], there have been few studies on the physiological mechanisms that affect the ETC in cotton, challenged by low P availability. In this study, even a slight shortage of P had a noticeable impact on photosynthetic electron transport. Low P availability affected the I-phase in the ETC, based on a study of ChlF OJIP transients. It is possible to use the change in fluorescence-transient curvature to measure the bioactive amount of P in plants. The objectives of this study were as follows: (1) To examine the effect of low P availability on growth parameters, antioxidant-enzymatic activities, photosynthetic parameters, Chl content, ChlF parameters, PEPC activity, and the contents of ATP and NADP(H) among different low-P-tolerant genotypes. (2) To explore the correlation between physiological indexes and PPUE under low P availability. The results of this study will help to better understand the possible influence of the mechanism of low P availability on cotton growth and development, photosynthesis, and antioxidant system, and provide a theoretical and physiological basis for the growth and tolerance of cotton under low-P-availability conditions. 

## 2. Materials and Methods

### 2.1. Materials and Growth Conditions of Plants

Based on our unpublished data, four cotton genotypes were used, including BX014 and LuYuan343 (LP tolerant), FJA and DLNTDH (LP sensitive). Hydroponically cultured plants were grown at the Cotton Research Institute of the Chinese Academy of Agricultural Sciences, Anyang, China.

Healthy seeds were surface sterilized with ethanol (70% v/v) for 10 min and disinfected with 3% sodium hypochlorite (v/v) for 20 min. The seeds were then rinsed five times with distilled water. Both cotton genotypes were incubated in a growth chamber in a combination of sand and vermiculite (w/w). This study was conducted using nutrient-solution hydroponics. The seedlings were transplanted into 8 L plastic boxes after germination. The plants were grown under natural light in a greenhouse at 25/20 °C (day/night) temperatures and 60% humidity. Each genotype was sown in seven replicates for each P treatment, each plant as a repeat. The seedlings were treated with various nutrient supplies at the two true-leaf stages. After transplantation, the seedlings were provided with 1/2-strength Hoagland solution for one week, followed by full-strength Hoagland solution until the end of the experiment. The plants were provided with 100 mL of dH_2_O daily, to replenish the water lost by transpiration. Each plant’s location was changed randomly each week to counteract the impact of the positioning [31]. 

The conventional nutrient solution was as follows: 0.1 mmol/L EDTA·Fe·Na, 1 mmol/L MgSO_4_·7H_2_O, 2 μmol/L ZnSO_4_·7H_2_O, 46 μmol/L H_3_BO_3_, 4 μmol/L MnCl_2_·4H_2_O, 0.3 μmol/L CuSO_4_·5H_2_O, 0.12 μmol/L (NH_4_)_6_Mo_7_O_24_·4H_2_O, 2.5 mmol/L Ca (NO_3_)_2_·4H_2_O, and combinations of three different P concentrations, including 15 µM and 50 μM (low P) and 500 μM (normal P), supplied as KH_2_PO_4_ in hydroponic culture. The nutrient solutions were continuously aerated with an electric pump, the airflow rate was 2 L/min, and the pH was maintained at 5.8 ± 0.5. The nutrient solutions were changed every seven days and aerated. The whole test period was 28 days, and the plants showed obvious signs of P deficiency; seven plants from each treatment were collected for further physiological measurements.

### 2.2. Measurement Traits and Methods

#### 2.2.1. Measurements of the Leaf Area and Gas-Exchange Parameters

The gas exchanges were measured from 9:00 to 11:00, using an LI-6800 portable photosynthetic system (LI-COR, Lincoln, NE, USA) equipped with a CO_2_ control module and a red–blue LED-light source. The photosynthetic-photon-flux density of the leaf chamber was set to 700 μmol m^–2^ s^–1^. The airflow rate in the leaf chamber was set as 700 μmol s^–1^. The concentration of CO_2_ in the reference chamber was set at 400 μmol mol^–1^ for the measurements. The net photosynthetic rate (Pn), stomatal conductance (Gs), transpiration rate (E), intercellular CO_2_ concentration (Ci), and CO_2_ concentration in the leaf chamber (Ca) were simultaneously recorded from seven seedlings per treatment. 

The leaf used to measure the gas-exchange parameters were collected, promptly immersed in N_2,_ and stored at −80 °C. The leaves were positioned with the main veins cut out on A4 paper, and a 4 × 4 cm piece of green cardboard was added as a control. Leaf images were obtained with a digital camera (7D, Canon, Inc., Tokyo, Japan), and image software (Media Cybernetics, Silver Spring, MD, USA) was utilized to measure the leaf area.

#### 2.2.2. Chlorophyl-a-Fluorescence Measurements

The youngest fully developed leaves were used to collect ChlF transients using a Handy PEA chlorophyll fluorometer (Hansatech Instruments, King’s Lynn, UK). After 30 min of dark adaptation, the leaves were continuously illuminated with 3000 µmol (photons) m^−2^ s^−1^ of red light to induce a fast ChlF curve. The fluorescence transients were recorded for 10 s, using a PIN photodiode. The graphic representations of the transients were all double-normalized.

The OJIP fluorescence-induction kinetics were recorded using a Handy Plant Efficiency Analyser (PEA; Hansatech Instruments). All the measurements were conducted in triplicate. The OJIP-curve red-light treatment was applied, to create 3000 µmol (photons) m^−2^ s^−1^ for 2 s. F0, F_K_, F_J,_ and F_I_ represent the fluorescence intensity at 20 μs, 300 μs, 2 ms, and 30 ms, respectively. We also used the fast-fluorescence-curve OJIP to calculate the following: Vt = (Ft − Fo)/(Fm − Fo) for O-P standardization, W_OJ_ = (Ft − Fo)/(F_J_ − Fo) for O-J standardization, and W_OI_ = (Ft − Fo)/(F_I_ − Fo) for O-I standardization. The descriptions of the parameters and their calculation formulae are shown in Table S1.

#### 2.2.3. Measurement of Malondialdehyde and Assays of Antioxidant Enzymes

The content of MDA was determined by the thiobarbituric-acid reaction [32]. After the samples were weighed and ground to a powder in liquid nitrogen, a 0.5 g sample of frozen leaves was used to determine the activity of antioxidant enzymes. Moreover, 10 mL of 50 mmol·L^−1^ sodium phosphate buffer (pH 7.8) was added, which contained 1% of polyvinylpyrrolidone (PVP), 0.2 mmol·L^−1^ of EDTA, and 10 mmol·L^−1^ of magnesium chloride, and was then centrifuged at 10,000× *g* for 15 min at 4 °C as previously described, with modifications [33]. The activities of SOD (20220916), POD (20220929), CAT (20220902), and the content of MDA (20220915) were detected spectrophotometrically using Solarbio assay kits, following the manufacturer’s instructions (Solarbio, Beijing, China).

#### 2.2.4. Determination of ATP, NADP(H), and the Activity of Phosphopyruvate Carboxylase 

A total of 0.1 g of fresh samples was homogenized in TEA-EDTA-saturated phenol (phenol-TEA; 0.6% triethylamine-phosphoric acid, pH 8.0 and 1 mmol L^−1^ EDTA, added to saturation in the phenol). The homogenate was then assayed spectrophotometrically, using the ATP (20220921) content assay kit (Solarbio, Beijing, China), following the manufacturer’s instructions. A total of 0.1 g of fresh leaves were homogenized with a pestle with 1 mL of 50 mM Tris–HCl (pH 8.0). The homogenate was centrifuged at 8000× *g* for 15 min, at 4 °C. The activity of PEPC (20220929) and the content of NADP(H) (20220915) were determined with detection kits (Solarbio, Bejing, China), according to the manufacturer’s instructions.

#### 2.2.5. Measurements of P Concentrations and Use of Efficiency Traits

After they had been ground to a fine powder, dried samples of the leaves were weighed to approximately 0.12 g each, after digestion with H_2_SO_4_-H_2_O_2_ at 360 °C for 3 h. The concentrations of P were measured using a Bran + Luebbe Continuous-Flow AutoAnalyzer III (AA3; Bran + Luebbe GmbH, Norderstedt, Germany). The definitions of P-use efficiency (PUE) for the cotton genotypes grown at various P concentrations were also included, and calculated as previously described [34], with minor modifications:

P accumulation (PA) = the P concentration determined by multiplying the plant dry weight;

P-uptake efficiency (PUtE) = the plant dry weight divided by P concentration;

P-use efficiency (PUE) = PA divided by dry weight;

Photosynthesis-P-use efficiency (PPUE) = the ratio of the maximum photosynthetic rate per unit P. 

#### 2.2.6. Determination of the Contents of Chlorophyll and Carotenoids

The pigments were extracted from the leaves, and 0.5 g of the uppermost unfolded leaf was weighed, cut into pieces, and placed in 15 mL of a mixture of acetone and ethanol (*v/v*; 1:1). The leaf was kept at room temperature (25 °C) and incubated in the dark for 24 h. A UV–Vis spectrophotometer (UV-1280; Shimadzu, Kyoto, Japan) was used to determine the absorbance at 663, 645, and 470 nm. In addition, Chl a and b were used to compute the total Chl and the Chl a/b ratio [35], and the total carotenoid concentration was determined as previously described [35].

#### 2.2.7. Statistical Analysis

All the data were analyzed for normality (Kolmogorov–Smirnov test) and tested for homogeneity of variance using SPSS 24.0 (SPSS, Chicago, IL, USA). A two-way analysis of variance (ANOVA) was used to evaluate the data, and the means were separated using a Tukey’s test at 5% level of significance. The morphophysiological traits were used to calculate the correlation of relationships in Microsoft Excel 2021 (Redmond, WA, USA). The figures were drawn with GraphPad Prism 9.0 (GraphPad, San Diego, CA, USA). All the data were expressed as the mean ± standard error (SE) of seven replicates; *, **, and *** represent *p* ≤ 0.05, 0.01, 0.001, respectively, and ns stands for not significant.

## 3. Results

### 3.1. Gas-Exchange Parameters among the Cotton Genotypes in Response to Differences in the Availability of P

**Figure 1:**
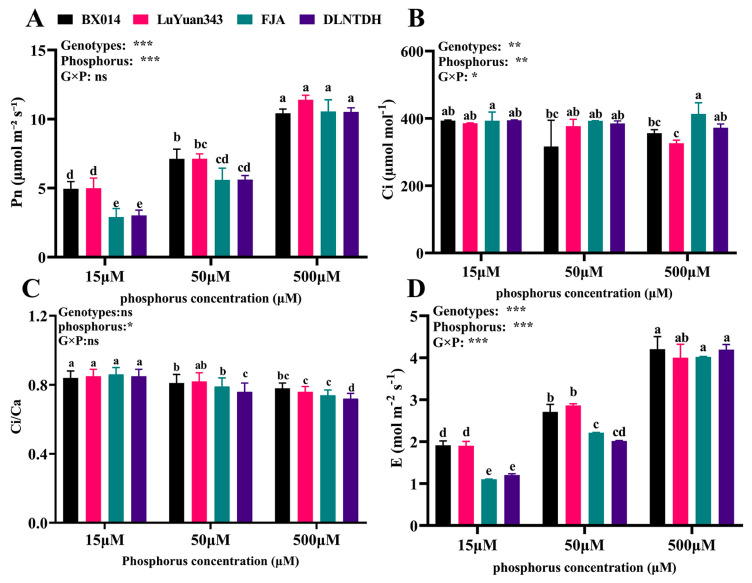
Effects of different P treatments on gas-exchange parameters. (**A**) Pn (net photosynthesis rate), (**B**) Ci (intercellular CO_2_ concentration), (**C**) Ci/Ca (ratio of intercellular-ambient-CO_2_ concentrations), and (**D**) E (transpiration-rate) content in the cotton genotypes, fully expanded barley leaves. The results are expressed as means **±** SE (*n* = 7). Statistically significant changes are indicated by different letters, using the two-way ANOVA and multiple-comparison test (*p* ≤ 0.05). *p*-values of the ANOVAs of genotypes (G), phosphorus level (P), and their interaction (G×P) are indicated as ns, not significant; *: *p* < 0.05, **: *p* < 0.01, ***: *p* < 0.001.

Different treatments of P dramatically impacted the parameters of leaf exchange of gases (Figure 1). Our results showed that the values for Pn, Gs, and E decreased in parallel with the level of P supplied (Figure 1). However, the extent of reduction differed among the cotton genotypes, with the responses of FJA and DLNTDH slightly lower than those of BX014 and LuYuan343 under conditions of low P (Figure 1). The Pn of BX014, LuYuan343, FJA, and DLNTDH were reduced by 52.47%, 56.16%, 72.47%, and 71.26%, respectively, under conditions of 15 μM P, and by 31.68%, 37.45%, 47.0% and 46.56%, respectively, under conditions of 50 μM P, compared with those of 500 μM P (Figure 1A, Table S3). The trend in the changes in E was similar to that in the Pn, and the effect of the low conditions of P was reduced (Figure 1D, Table S3). With the increase in P concentration in cotton leaves, the Ci/Ca ratio decreases, but there is no significant difference among genotypes (Figure 1C).

Notably, compared with conditions of 15 μM P, the Ci in BX014 and LuYuan343 decreased by 19.56%, 2.18%, 10.47%, and 18.22% in conditions of 50 µM and 500 μM P, respectively, but there was no difference among the different P treatments in FJA and DLNTDH (Figure 1D, Table S3). As expected, there was no discernible difference in gas-exchange parameters between these genotypes under 500 μM P (Figure 1).

### 3.2. Difference in the Growth Parameters among Cotton Genotypes in Response to Different Availabilities of P 

**Figure 2:**
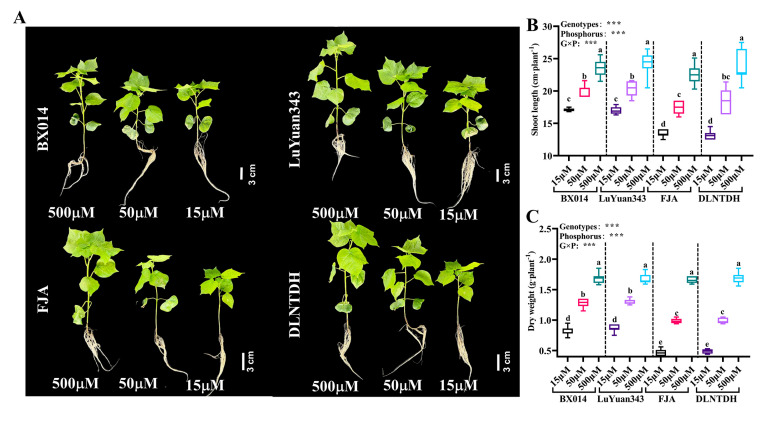
(**A**) Phenotypes of varieties with different P efficiency under different P treatments; (**B**) shoot length, and (**C**) dry weight at 28 days after various P treatments. The results are expressed as means **±** SE (*n* = 7). Statistically significant changes are indicated by different letters using the two-way ANOVA and multiple-comparison test (*p* ≤ 0.05). *p*-values of the ANOVAs of genotypes (G), phosphorus level (P), and their interaction (G×P) are indicated as ns, not significant; ***: *p* < 0.001.

The P treatments differentially affected the development of cotton seedlings (Figure 2A). With the decrease in P supply, the plant height and dry weight were drastically reduced. However, the degree of reduction varied in different genotypes (Figure 2A). 

Compared with 500 μM P, the shoot length of BX014 and LuYuan343 decreased by 29.41% and 28.31%, respectively, under 15 μM P, and by 17.18% and 14.07%, respectively, under 50 μM P (Figure 2B, Table S2). The shoot length decreased by 40.71% and 44.71%, respectively, under 15 μM P, and by 22.56% and 21.32%, respectively, under 50 μM P in FJA and DLNTDH, compared with the 500 μM conditions of P (Figure 2B, Table S2). The dry weight decreased by 50.59% and 47.90%, respectively, under15 μM P, and by 23.81% and 22.16%, respectively, under 50 μM P in BX014 and LuYuan343, compared with 500 μM P (Figure 2C, Table S2). The dry weight of FJA and DLNTDH decreased by 71.69% and 71.01%, respectively, in 15 μM conditions of P, and by 40.36% and 42.01%, respectively, under 50 μM conditions of P, as compared with 500 μM conditions of P, (Figure 2C, Table S2). In particular, the shoot length and dry weight of the LP-sensitive genotypes (FJA and DLNTDH) decreased considerably more than those of the two LP-tolerant genotypes (BX014 and LuYuan343) in both conditions of low P.

### 3.3. Physiological Differences among Cotton Genotypes in Response to Different P Availability

**Figure 3:**
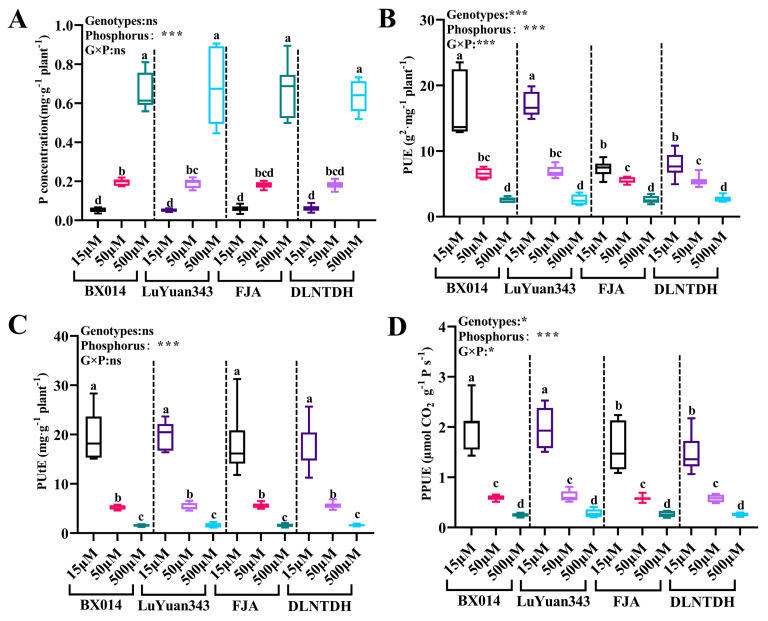
Changes in (**A**) P concentration, (**B**) PUE (P-use efficiency), (**C**) PUtE (P-uptake efficiency), and (**D**) PPUE (Photosynthetic P-use efficiency) in cotton, under different treatments. The results are expressed as means **±** SE (*n* = 7). Statistically significant changes are indicated by different letters using the two-way ANOVA and multiple-comparison test (*p* ≤ 0.05). *p*-values of the ANOVAs of genotypes *(G)*, phosphorus level (P), and their interaction (G×P) are indicated as ns, not significant; *: *p* < 0.05, ***: *p* < 0.001.

There were distinct differences in PA, PC, PUE, and PUtE leaf area, and in PPUE among the four genotypes, in response to P treatment (Figure 3). In comparison with the 500 μM condition of P, all the genotypes that were treated with 15 µM and 50 μM P accumulated lower concentrations of P (Figure 3A and Figure S1A). Overall, there was little genotypic variation regarding P concentration. The genotypes that were tolerant to low LP had significantly (*p* ≤ 0.05) higher parameters than the LP-sensitive genotypes under the 15 µM and 50 μM conditions of P (Figure 2A,B). In contrast, in comparison with 500 μM P, the PUE increased 5.24-, 5.57-, 2.31-, and 2.12-fold at 15 μM P, and 1.52-, 1.63-, 1.09- and 1.02- fold under 50 μM P, in BX014, LuYuan343, FJA, and DLNTDH, respectively (Figure 3B, Table S2). Simultaneously, the PUtE increased 11.58-, 11.80-, 10.59-, and 9.80- fold under 15 μM conditions of P, and 2.31-, 2.41-, 2.52-, and 2.31-fold under 50 μM conditions of P in BX014, LuYuan343, FJA, and DLNTDH, respectively, as compared with 500 μM conditions of P (Figure 3C, Table S2).

### 3.4. Contents of Chlorophyll Pigments among Cotton Genotypes in Response to Different Availabilities of P

Both the 15 and 50 μM conditions of P reduced the leaf area of all the cotton genotypes (Figure S1B, Table S2). Compared with FJA, DLNTDH, BX014, and LuYuan343, the leaf areas were reduced less under the 15 µM and 50 μM conditions of P (Figure S1B, Table S2). As shown in Figure 2, the leaf area decreased by 34.52%, 34.59%, 47.64%, and 46.62% under the 15 μM conditions of P and by 20.30%, 19.54%, 29.51%, and 29.76% under the 50 μM conditions of P in BX014, LuYuan343, FJA, and DLNTDH, respectively, compared with the conditions of 500 μM P (Figure S1B, Table S1). In contrast, the values for the PPUE of BX014, LuYuan343, FJA, and DLNTDH increased 6.91-, 6.18-, 4.96-, and 4.70-fold, respectively, under the 15 μM conditions of P, and 1.37-, 1.23-, 1.23- and 1.24-fold, respectively, under the 50 μM conditions of P, compared with those under conditions of 500 μM P (Figure 3D, Table S2). However, there was no variation in the PPUE among these genotypes when the conditions were 50 µM and 500 μM P.

The contents of Chl a in BX014, LuYuan343, FJA, and DLNTDH decreased by 50.99%, 49.63%, 29.73%, and 27.11%, respectively, under conditions of 15 µM, and by 29.62%, 32.61%, 6.76%, and 11.11%, respectively, under conditions of 50 μM P, compared with those of 500μM P (Figure S2A, Table S3). The contents of Chl b in these genotypes under conditions of 15 µM and 50 μM P also decreased, compared with the conditions of 500 μM P. Notably, the contents of Chl a and b among these genotypes did not differ under conditions of 500 μM P (Figure S2A,B). 

Simultaneously, a reduction in Chl, carotenoids, and Chl /carotenoid in all the genotypes was observed under 15 µM and 50 μM P, compared with those at 500 μM. However, the ratio of Chl a/b among these genotypes did not differ from each other under all the P treatments (Figure S2C–F). Notably, the contents of pigments indicators, except for Chl a/b in the two LP-tolerant genotypes BX014 and LuYuan343, were greater than those of the two LP-sensitive genotypes FJA and DLNTDH by a substantial margin, under low conditions of 15 µM and 50 μM P (Figure S2).

### 3.5. Genotypic Variation in Antioxidant Activities and the Content of MDA in Response to Different Availabilities of P

**Figure 4:**
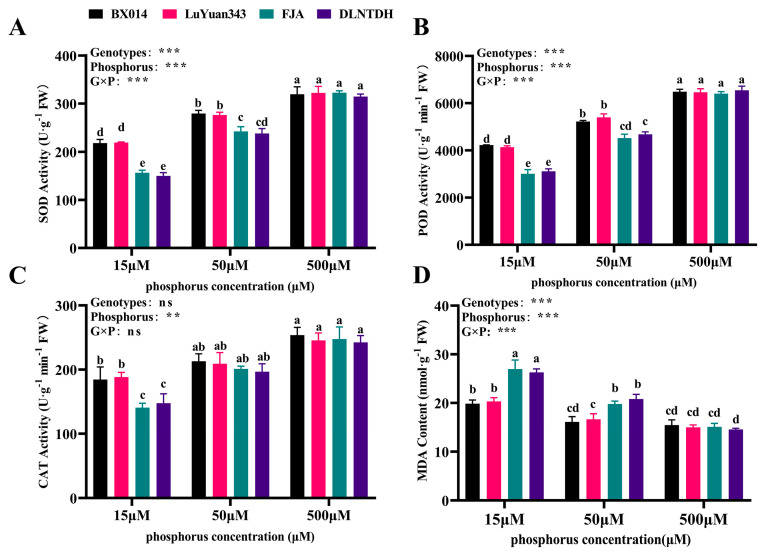
Various P treatments’ effects on antioxidant substances. (**A**) SOD (superoxide-dismutase) activity, (**B**) POD (peroxidase activity, (**C**) CAT (catalase) activity, and (**D**) MDA (malondialdehyde) content in the cotton genotypes, fully expanded barley leaves. The results are expressed as means ± SE (*n* = 7). Statistically significant changes are indicated by different letters, using the two-way ANOVA and multiple-comparison test (*p* ≤ 0.05). *p*-values of the ANOVAs of genotypes (G), phosphorus level (P), and their interaction (G×P) are indicated as ns, not significant; **: *p* < 0.01, ***: *p* < 0.001.

In our study, low conditions of P reduced the activities of CAT, POD, and SOD in the cotton leaves (Figure 4). The activity of SOD, BX014, LuYuan343, FJA, and DLNTDH decreased by 51.56%, 52.38%, 24.91%, and 24.36%, respectively, under conditions of 15 µM P, and by 24.91%, 24.36%, 12.55%, and 14.22%, respectively, under conditions of 50 M P, compared with those of 500μM P (Figure 4A, Table S4). The activity of POD in the different P treatments showed a similar trend, which was a decrease, with a reduction in the concentration of P (Figure 4B). In comparison with the conditions of 500 μM P, the activity of CAT decreased remarkably, by 27.29%, 23.24%, 48.24%, and 39.08% in BX014, LuYuan343, FJA, and DLNTDH, respectively, under conditions of 15 μM P, but there was no variation or difference among these genotypes under conditions of 50 μM P (Figure 4C, Table S4). However, compared with conditions of 500 μM P, those at 15 μM increased the content of MDA by 28.41%, 35.66%, 78.49%, and 80.47% in BX014, LuYuan343, FJA, and DLNTDH, respectively, while conditions of 50 μM P also increased it, by 31.10% and 43.04% for FJA and DLNTDH, respectively; however, BX014 and LuYuan343 did not differ in the conditions of 50 µM and 500 μM P (Figure 4D, Table S4).

### 3.6. The Contents of ATP and NADP(H) and PEPC Activity among Cotton Genotypes in Response to Different Availabilities of P

**Figure 5:**
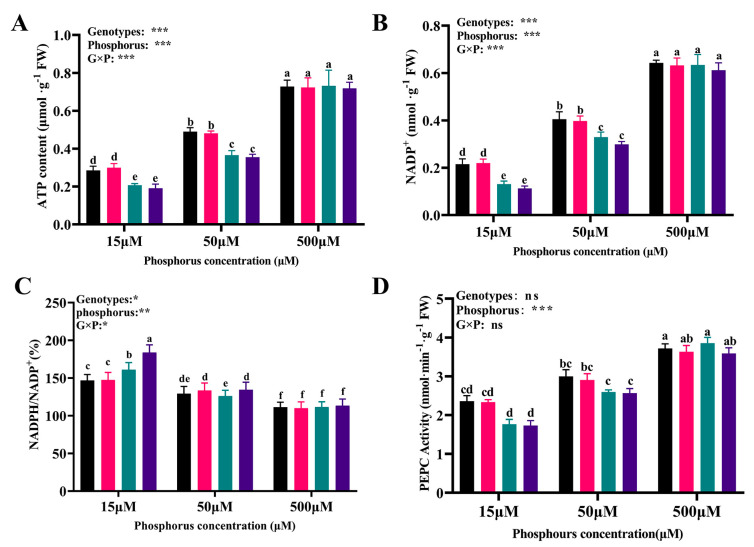
Effects of different P treatments on the availability and quantity of photosynthesis-related enzymes. (**A**) ATP (adenosine-triphosphate) content, (**B**) NADP^+^ (nicotinamide-adenine- dinucleotide-phosphate) content, (**C**) NADPH/NADP^+^, and (**D**) PEPC (phosphopyruvate-carboxylase) activity in the cotton genotypes, fully expanded barley leaves. The results are expressed as means **±** SE (*n* = 7). Statistically significant changes are indicated by different letters, using the two-way ANOVA and multiple-comparison test (*p* ≤ 0.05). *p*-values of the ANOVAs of genotypes (G), phosphorus level (P), and their interaction (G×P) are indicated as ns, not significant; *: *p* < 0.05, **: *p* < 0.01, ***: *p* < 0.001.

The results showed that the content of ATP in BX014, LuYuan343, FJA, and DLNTDH decreased by 60.85%, 58.54%, 71.70%, and 73.42%, respectively, under conditions of 15 μM P, and by 32.62%, 33.50%, 50.02%, and 51.30%, respectively, under conditions of 50 μM P, (Figure 5A, Table S4). Similarly, the low conditions of P decreased the content of NADP^+^ in the LP-tolerant genotypes, compared with those of the genotypes subjected to conditions of 500 μM P (Figure 5B). 

### 3.7. The Fluorescence-Increase-Kinetics OJIP Curves among Cotton Genotypes in Response to Different Availabilities of P

The ratio of NADPH/NADP^+^ in BX014, LuYuan343, FJA, and DLNTDH increased by 31.75%, 34.07%, 44.28%, and 62.21%, respectively, under conditions of 15 µM P, and by 15.98%, 21.44%, 13.06%, and 18.62%, respectively, under conditions of 50 M P, compared with those of 500 μM P (Figure 5C). Compared with conditions of 500 μM P, the activity of PEPC decreased dramatically, by 36.58%, 35.72%, 54.22%, and 51.69%, and 19.30%, 19.98%, 32.72%, and 28.45% with respect to BX014, LuYuan343, FJA, and DLNTDH, respectively, under conditions of l5 µM and 50 μM P, respectively. (Figure 5D, Table S4).

**Figure 6:**
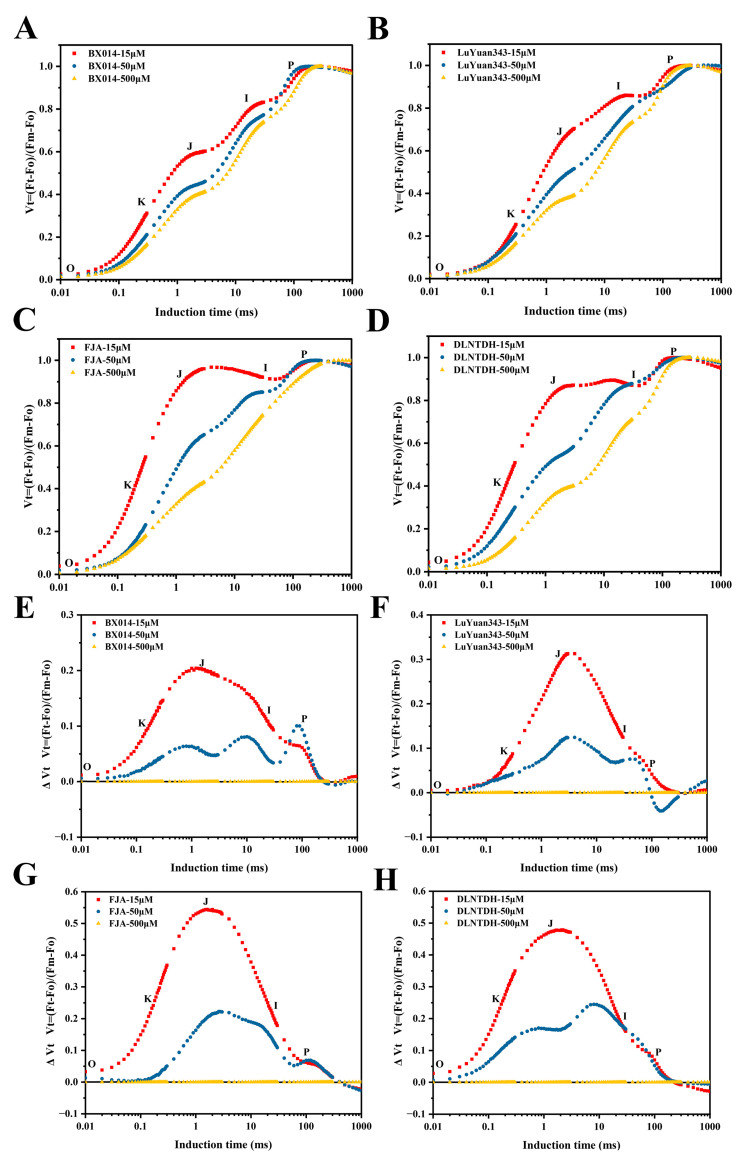
Variations in OJIP-kinetics ChlF rise of cotton leaves after different P treatment. ((**A**) BX014, (**B**) LuYuan343, (**C**) FJA, and (**D**) DLNTDH, Vt = (Ft − F0)/(Fm − F0)), and the comparative variations of ChlF ((**E**) (BX014), (**F**) (LuYuan343), (**G**) FJA, and (**H**) DLNTDH, ∆Vt = Vt (treatments of 15 μM and 50 μM) − Vt (treatments of 500 μM) in each genotype), third row of leaves, fully developed. Each curve represents the seven replicate averages.

To more clearly observe how P treatments affect transient dynamics, the curves have been represented on the graph based on the relative variable fluorescence, Vt = (Ft − Fo)/(F_M_ − Fo) (Figure 6). All the genotypes in this study showed a different typical polyphasic rise in the OJIP-transient curve under conditions of 15 µM and 50 μM P (Figure 6). A recognizable increase in the O-J-I phase in the OJIP-transient curve was observed in FJA and DLNTDH under conditions of 15 µM and 50 μM P, but as the concentration of P increased, this increase was observed to decline (Figure 6C,D). The application of 15 µM and 50 μM P also increased the J-I-P phase of the OJIP-transient curve in BX014 and LuYuan343 (Figure 6A,B). However, the rate of increase in the OJIP-transient curve represented by the genotypes sensitive to LP was higher than those tolerant to LP (Figure 6).

Calculating the relative variable fluctuations in the fluorescence curve enabled researchers to determine changes in the increase in OJIP-fluorescence kinetics. They were created by deducting the values of normalized fluorescence (between O and P) in all the genotypes (ΔV = Vt [15 µM and 50 μM P] − Vt [500 μM P]) (Figure 6). Positive ΔVt values indicated lower rates of electron transportation, i.e., with negative values. The electron transport efficiency (ETE) decreased. At conditions of 15 μM P, a positive ΔK (at 0.3 ms), ΔJ (at 2 ms), and negative ΔI (at 30 ms) were established, and then turned into positive ΔP peaks on BX014 and LuYuan343 at 100 ms (Figure 6E,F). The curves under conditions of 50 µM P in these genotypes changed in a manner similar to those under conditions of 15 µM P, but the amplitude was less than those in the 15 μM conditions of P (Figure 6E,F). At the 15 μM P conditions of FJA and DLNTDH, a positive ΔK was created at 0.15 ms and a ΔJ peak at 2 ms; the value then turned to produce a negative ΔI peak at 30 ms, and the value was also negative in ΔP (Figure 6G,H). There was a similar trend in the conditions of genotypes sensitive to LP at 50 μM P, but the amplitude was less than that at conditions of 15 μM P (Figure 6G,H). These findings demonstrated a disruption in the photosynthetic-electron-transport cycle under low-P conditions in which the LP-tolerant genotypes could retard this damage, in contrast to the LP-sensitive genotypes.

### 3.8. The O-J-I-P Phase among Cotton Genotypes in Response to Different Availabilities of P

Additional normalizations and associated subtractions were made to assess the events captured by the OK, OJ, OI, and IP phases (difference kinetics). In addition, the fluorescence-increase-kinetics curves are shown (Figure S3). The fluorescence-increase-kinetics data of different P treatments were double-normalized by O (20 μs) and K-step (300 μs) to show the L-band as W_OK_ and ΔW_OK,_ as described in Table S1. The unit groupings of PSII or the energetic connectedness between the antenna and PSII RCs are known to be indicated by the L-band. It was observed most clearly that at the 50 μM conditions of P the L-band increased, and the conditions of 15 µM and 500 μM P slightly increased the L-band of all the genotypes (Figure S3B). It was clear that the W_L_ and ΔW_L_ values of FJA and DLNTDH increased under the 15 µM and 50 μM conditions of P, but those of BX014 and LuYuan343 did not increase, compared with the 500 μM conditions of P (Figure S3B). This suggested that low-P conditions caused an increase in the L-band, owing to the increase in the J-step (Figure S3B).

The increases in the ChlF kinetics were standardized to ensure that the effects of varied P treatments on the K-step were as expected by the O-step (20 μs) and J-step (2 ms), and are shown in Figure S3C. W_OJ_ and ΔW_OJ_ are described in Table S1. The curves of ΔW_OJ_ indicated that low conditions of P induced the occurrence of the K-band, particularly for FJA and DLNTDH (Figure S3C). Only the conditions of 15 and 50 μM P increased the value of W_K_ (Figure S3A), which corroborated the fact that low conditions of P can indeed damage some of the operating OEC facilities.

In addition, the fluorescence-kinetics curves between the O-step and I-step (30 ms) were standardized, and W_OI_ and ΔW_OI_ are also described in Table S1, as well as W_OI_ ≥ 1, which was plotted in the linear 30–270 ms time range, to show the IP phase (Figure S3D). ΔW_OI_ was altered to show the effects of different P treatments on the J-step, and the results were similar to those of ΔVt (Figure S3D). The 15 µM and 50 μM conditions of P decreased the amplitude of W_OI_ curves to various P treatments (Figure S3D). Another fluorescence-increase-kinetics normalization, W_IP_, (normalized by the I- and P-step) is presented in Figure S3E.

The time point at W_IP_ = 0.5 (half-time of the increased curves) can be used to reflect the rate of reduction of PSI and the pool of electron acceptors. It is clear that the half-rise time values of 15 μM P (approximately 90 ms) and 50 μM P (approximately 70 ms) of BX014 and LuYuan343 were greater than those of FJA and DLTDH at the same treatments of P (Figure S3E). This suggests that low-P conditions lower the end-electron-acceptor reduction rates of PSI, and the LP-sensitive genotypes gave higher results than the LP-tolerant genotypes.

### 3.9. The JIP-Test Equations Produce the PSII Biophysical Parameter

**Figure 7:**
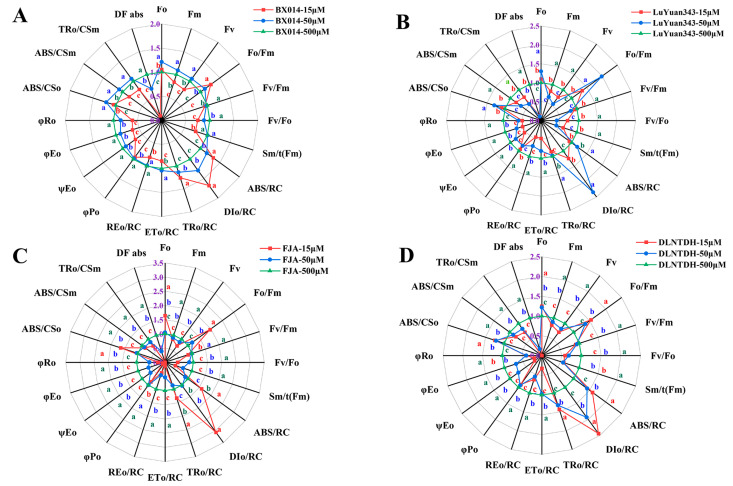
Spider plot of Chl a fluorescence of cotton leaves, the value of 500μM P set 1. (**A**) BX014, (**B**) LuYuan343, (**C**) FJA, (**D**) DLNTDH, at different P treatments. The results are expressed as means ± SE (*n* = 7). Statistically significant changes are indicated by different letters, using the one-way ANOVA and multiple-comparison test (*p* ≤ 0.05).

All the fluorescence parameter values were standardized to the values in the radar graphic that represents the JIP-Test parameters (Figure 7). The majority of the values of parameters that define PSII functioning are depicted in the spider plot, and show how all the cotton genotypes responded to low conditions of P, compared with those at 500 μM P (Figure 7). The nutritional stress of plants can be evaluated using Fv/Fm, which reflects the maximal quantum yield of the primary photochemistry of PSII. The Fv/Fm of each genotype decreased dramatically under 15 µM and 50 μM P, compared with those at 500 μM P, but values for the LP-tolerant genotypes were higher than those of the LP-sensitive genotypes at conditions of 15 µM and 50 μM P (Figure 7, Table S5). 

The absorption flux, trapped-energy flux, and electron-transport flux per reaction center are denoted as ABS/RC, TRo/RC, and DIo/RC, respectively. There were increases in ABS/RC, TRo/ RC, and DIo/RC under the conditions of 15 µM and 50 μM P, compared with those at 500 μM P (Figure 7, Table S5). The flux model shows the change in energy flux (Figure S4). In addition, the electron flow per active reaction center (REo/RC) decreased under 15 and 50 μM P, compared with the conditions of 500 μM P of the LP-sensitive genotypes. In contrast, the electron transport per reaction center (ETo/RC) of BX014 and LuYuan343 decreased slightly under conditions of 15 μM P, and the distribution of energy became more balanced (Figure 7, Figure S4). Conditions of 15 and 50 μM P had the lowest specific-energy-flux dissipation in each genotype, and higher specific-energy-flux transfer to electron acceptors was used to achieve the maximum electron flux at the end of PSI (Figure S4).

φPo, φEo, and ΨEo are the ratios beyond the QA-triggered electron transfer. More electron acceptors in the electron transport chain by exciton occupancy in the excitons are caught by the reaction center, which enables it to intuitively represent the light-energy-transfer efficiency of the PSII reaction center. There were reductions in these parameters under conditions of 15 µM and 50 μM P, compared with those in the 500 μM P conditions of FJA and DLNTDH, whereas those in BX014 and LuYuan343 decreased slightly (Figure 7, Table S5).

### 3.10. Relationship between PPUE and Morphophysiological Variables

**Figure 8:**
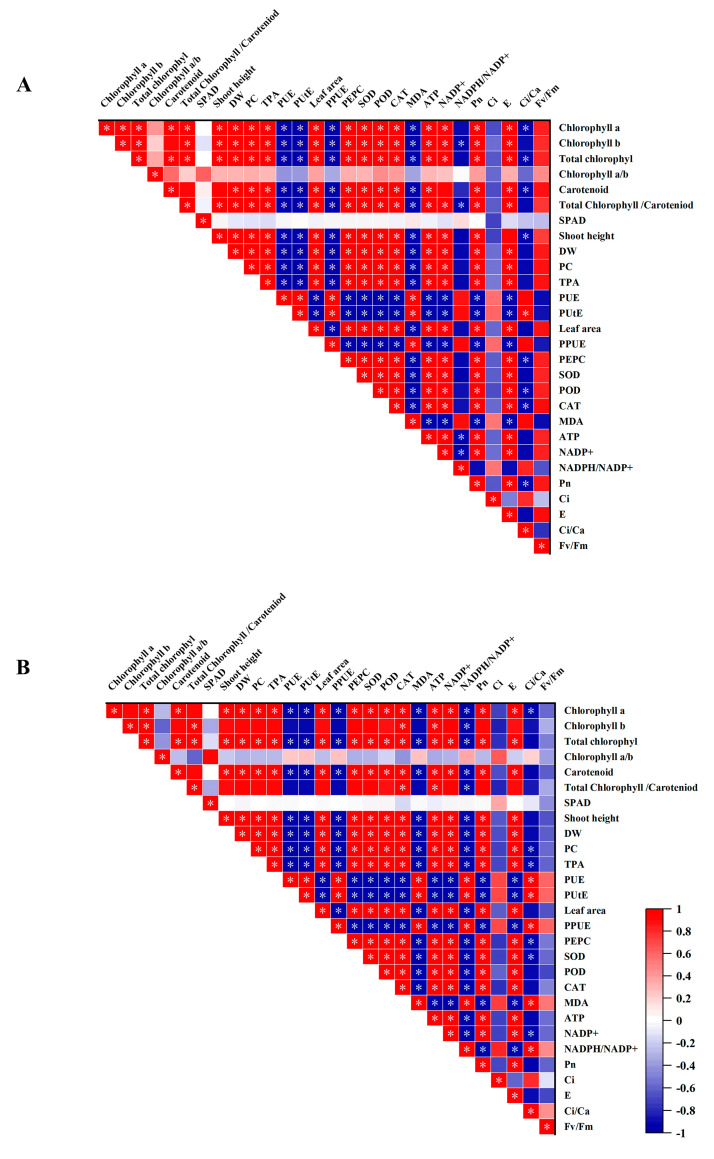
Correlation analysis among measurement indicators in the (**A**) LP-sensitive genotypes and (**B**) LP-tolerant genotypes. Asterisk shows significant differences at *p* < 0.05. Chl a,b (Chl a,b), net photosynthetic rate (Pn), intercellular CO_2_ (Ci), the ratio of intercellular-ambient-CO_2_ concentrations (Ci/Ca), and transpiration rate (E), the maximal quantum efficiency of PSII photochemistry (Fv/Fm), P concentrations (PC), P accumulation (TPA), P-use efficiency (PUE), P-uptake efficiency (PUtE), photosynthetic-P-utilization efficiency (PPUE), SPAD (SPAD value), dry weight (DW), superoxide dismutase (SOD), peroxidase (POD), catalase (CAT), malondialdehyde content (MDA), phosphoenolpyruvate carboxylase (PEPC) adenosine triphosphate (ATP), nicotinamide adenine dinucleotide phosphate (NADP^+^), and nicotinamide adenine dinucleotide phosphate (NADP). *: *p* < 0.05.

To better understand the relationship between the morphological and physiological parameters of cotton seedlings and the key parameters of the ETC under low availabilities of P, we analyzed the correlation between parameters in this study (Figure 8). Our results showed that the cotton genotypes positively correlated in the PUE and PPUE (Figure 8). In addition, the antioxidant substances had a strong positive correlation with the growth parameter of cotton seedlings and a negative correlation with PUE and PUtE. There was a negative correlation between Pn with PUE, PUtE, and PPUE, in all genotypes (Figure 8). It is worth noting that there is a positive correlation between antioxidant-enzyme activity and gas-exchange parameters with dry weight. There is also a positive correlation between photosynthetic pigment and dry matter. Pearson’s correlation analysis revealed that antioxidant-enzyme activity, photosynthetic pigment, and Pn were significantly high and positively correlated.

## 4. Discussion

**Figure 9:**
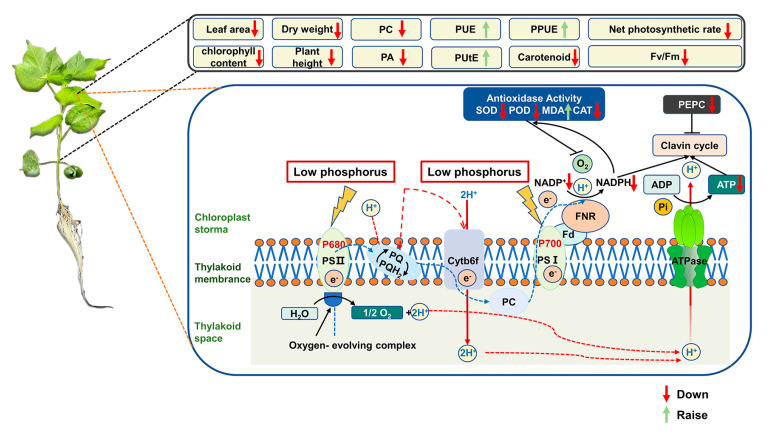
Pictorial representations of photosynthetic-electron-transport changes and feedback mechanisms responsible for low P availability. Processes that appear to be down-regulated are indicated by red arrows, while green arrows indicate processes that appear to be up-regulated. Unit size and font size represent the activities of the organelle components.

Our results show that P availability is a main factor that limits cotton growth, which is consistent with other studies [1]. In this study, the availability of low P affected the physiological and biochemical parameters of four cotton genotypes, and this change is described in (Figure 9). Previous studies have shown that a shortage of P prevents photosynthesis [8]. However, little is known about the effects of low P availability on P absorption, photosynthesis, and the antioxidant system in cotton.

### 4.1. Effects of the Low Availability of P on the Enzymes Related to the Photosynthesis System

Photosynthesis commonly declines under P deficiency, since P is involved in several cellular processes, including energy conservation, metabolic regulation, and signal transduction [8]. In this study, Pn, Gs, and E decreased under low P conditions. Nevertheless, an increase in Ci in this result demonstrates a clear dawn inhibition of photosynthesis, due to the lack of P carriers, strongly evidencing that the stomatal limitation is not imposing any barrier to the entry of CO_2_. Previous studies have shown that the reduction in the stomatal conductance limits the diffusion of CO_2_ necessary for carboxylation reactions, thus reducing Pn [36]. 

A P shortage has previously been demonstrated to immediately affect the ETC, because of its crucial roles [8]. Based on our results, low P inhibited the contents of ATP and NADP(H) in cotton leaves. A previous study also found that low P reduces the contents of ATP and NADP(H) in barley [8]. Low P stress reduces the ETC protein complex, including PSII, Cytb6f, and PSI [37]. As a response, a deficiency in P limited photosynthesis by decreasing the efficiency of ETC and that of ATP, NADP(H), and biosynthesis [38]. These studies demonstrated that ATP-synthase activity was controlled in response to metabolic or physiological circumstances, which enabled control over assimilatory responses [39]. The levels of NADP^+^ serve as a substrate for the synthesis of NADP(H). In this study, their levels were also dramatically reduced by a shortage of P, which demonstrates that a larger proportion of NADP^+^ is still in the reduced form, NADP (H), owing to its inability to be used in the Calvin cycle because of ATP limitations and greater PSI activity [38]. The C3-type PEPC exists in all plants, and participates in many physiological functions, such as the supply of intermediates in the tricarboxylic-acid cycle, the synthesis of OAA, and the subsequent synthesis of malic acid and its derivatives [40]. Relevant research on cotton found that PEPC enzyme activity is conducive to the synthesis, transportation, and decomposition of photosynthetic products, promoting fiber development and improving yield [41,42]. The activity of PEPC in this study was significantly (*p* ≤ 0.05) reduced by a deficiency of P. The growth of -P plants has been reported to be enhanced by overexpressing PEPC in rice and citrate synthase in cotton [43]. Our results demonstrate that the decrease in PEPC activity under low-P conditions could be one of the important limiting conditions for reducing the photosynthetic rate.

Severe P deficiency or toxicity can alter the photosynthetic apparatus and photosystems, thus causing photo-oxidative stress [38]. As is well-known, the early stages of photosynthetic reactions are strongly related to ChlF. Changes in the fundamental responses that alter PSII are effectively represented by the ChlF parameter, which serves as an effective index when the plant is under stress [44]. A potent non-invasive technique for tracking changes in photochemical efficiency and photosynthetic electron transport is the measurement of ChlF transients [45]. Depletion of the I-step from the fast OJIP portion of the ChlF transient can be used to identify a shortage of P [46]. The reaction can be used to precisely forecast the bioavailable P in both monocots and dicots, since it is so sensitive to the P content in leaf tissue [47]. Our study of cotton genotypes showed a difference in typical OJIP-transient and ChlF measurements among different availability conditions of P. It is possible that the higher Fo, rather than the decreased fluorescence, caused the decreased IP phase in P-deficient leaves from t = 30 ms to t = 300 ms (I step to P step). This showed that the plastocyanin had degraded, and the electrons were transferred from the FeS clusters to ferredoxin. Similar findings were also observed under a P deficit in barley and pomelo [48]. An impeded electron flow from the OEC to the secondary electron donor of PSII can be used to explain the positive K-band, and a decrease in the turnover of QA subsequently slowed [49]. Our results showed that at approximately 0.15 ms, a positive L-band was visible, indicating that in P-deficient leaves, the PSII units were less clustered or that the movement of energy between independent PSII units was hampered. A similar result has also been previously described [48].

Our findings showed that cotton genotypes under low-P stress had lower Fv/Fm values than those under normal P condition. As a result, the P shortage impacted the PSII-response centers. A previous study showed that, owing to the feedback inhibition of photosynthesis, a severe P deficit reduces the Fv/Fmand results in Chl loss [23]. Our results showed that, despite the higher photon-energy-capture RC and CSo under conditions of P deficiency, as shown by the increase in ABS/RC, DIo/RC, and TRo/RC, a lack of P reduced the efficiency of electron transport from the donor side of PSII to the final acceptor side of PSI. This was revealed by a decrease in ψEo, φEo, and φRo, which led to reduced CO_2_ assimilation in cotton genotypes deficient in P. Similarly, in wheat leaves, reduced energy trapping and electron transport per excited cross section were observed with increasing salt concentrations, indicating a decreased energy-absorption efficiency of PSII [50]. Some previous studies showed impaired electron transport from the donor side of PSII to the final acceptor side of PSI when P is lacking [8].

### 4.2. Effects of the Low Availability of P on the Enzymes Related to the Antioxidant System

Photosynthesis involves the conversion of sunlight energy into stored chemical energy, which is realized through the electronic transmission of a series of oxidation–reduction reactions. Due to the risk of molecular-oxygen reduction, excessive photosynthetic electron transport may be dangerous, resulting in excessive accumulation of ROS [51]. To avoid excessive ROS generation, it is necessary to coordinate the electron-transfer rate with the capacity of the electron acceptor in the chloroplast matrix. The imbalance between the donor and the receptor sides of PSI can lead to inactivation, which is called PSI photoinhibition. Therefore, the instantaneous or continuous generation of ROS may occur. The photosynthetic membrane protein complexes (PS II, the b6/f complex, and PS I) can produce ROS under specific conditions. Possible sources of ROS in PS II and PS I are the Chl-containing, light-harvesting arrays in both photosystems. ROS can oxidatively damage proteins, pigments, lipids, and nucleic acids [52]. A previous study showed that low P levels could also induce ROS production by compromising the use of light energy harvested by reaction centers and synthesizing ATP and NADPH [53], which results in electron-transport-chain overload [44].

Previous studies have shown that a deficiency or excess of macronutrients can cause the overproduction of ROS in plants and rapidly trigger the oxidative defense reaction [54]. When the stress reaches a certain intensity, the balance between ROS and the antioxidant system is broken. Antioxidant substances are widely found in aerobic or oxygen-resistant organisms. They have been proven to catalyze the reaction of superoxide anions to reduce or eliminate excessive ROS produced during metabolic processes [38]. In this study, the conditions of 15 µM and 50 µM P substantially reduced the activities of SOD, POD, and CAT. Previous research has shown that the activity of ROS-scavenging enzymes increases in response to low-P stress, to shield chloroplasts from the photooxidative damage induced by low P and to maintain redox homeostasis [44]. Some studies found that the enzyme activities of SOD, POD and CAT would decrease under a low-P environment, which is consistent with our results [26,28]. 

The MDA is the production of lipid peroxide inside cells. Its content could reflect the degrees of the lipid peroxide of the organism, and be enhanced under P-deficiency stress [55]. A previous study showed that a P shortage could result in increased contents of MDA in the stems of lentils (*Lens culinaris* Medik.) [56]. Our results showed that low P availability increased the MDA content of leaves, which indicated that low P availability aggravated the lipid peroxidation of plant membranes. However, the MDA content of low-P-tolerant genotypes was lower than that of low-P-sensitive genotypes. Therefore, the amount of MDA may be a response indicator of the physiological mechanism of the low-P-tolerant genotypes to adapt to low P availability.

### 4.3. Low Availability of P Affects Growth Parameters, Phosphorus Utilization, and PPUE

Photosynthesis is fundamental for plant growth and total-biomass production. Manipulating photosynthetic performance can optimize growth and biomass. Therefore, the photosynthesis condition is considered the major factor influencing yield in modern agricultural production [57]. Plant photosynthetic capacity depends on the net photosynthetic rate and photosynthetic area of the plant [58]. Chl content, especially Chl b content, and stomatal conductance are key factors that greatly affect the Pn [59]. A previous study showed that P deficiency caused stunted growth and resulted in early leaf senescence, with severely decreased leaf area and photosynthesis [60].

In this study, cotton seedlings cultured under P-deficient conditions were dwarfed, with low pigment content. The reason may be that both ROS generated by P deficiency promote Chl degradation and limit photosynthesis and cotton growth. Our studies have demonstrated a significant positive correlation between P efficiency and photosynthetic-related traits in cotton genotypes, particularly under low-P conditions. We have also observed that leaves differed greatly in morphology under low-P conditions, with leaves grown under P deficiency showing a smaller leaf area compared with those treated with normal P; this is consistent with other research [61], and this result suggests that P deficiency may decrease photosynthesis and ultimately affect plant growth and biomass. These findings were also observed in other studies [62]. In this study, the dry weight decreased under low-P-availability conditions. The lower dry weight may be caused by the decrease in Chl content, which is consistent with previous studies [63]. In addition, there was a significant correlation between Chl a content and dry weight of cotton leaves and the relevant fluorescence parameters (Figure 8). This suggests that ChlF parameters are to some extent a response to the growth status of the plant.

An increasing amount of research shows that achieving relatively fast rates of photosynthesis with high PPUE requires a delicate balance in investment among foliar-P fractions [64]. For instance, some plants suffering from P deficiency can decrease their overall requirement for foliar P by reducing investment in non-metabolite-P fractions to different degrees, thus buffering the direct P restriction of photosynthesis [11]. Plant species, and even genotypes of certain species, differ in their ability to maintain their photosynthetic activity under P limitation. Significant interspecific and intraspecific variation exists for PPUE under low-P stress [11]. However, our understanding of the biochemical basis for differences in PPUE among and within species is still in its infancy. This study showed that the cotton seedling PPUE under low-P conditions was higher than that under normal treatment, and the cotton genotypes with low-P tolerance showed higher PPUE under low-P conditions. We also found a significant negative correlation between PPUE and P content in leaves. This increases the PPUE under low-P conditions.

In our study, the concentrations of leaf P in the different cotton genotypes were impacted differently by low P. Previous studies have demonstrated that, compared with the low-P-sensitive cotton genotypes, the low-P-tolerant cotton varieties improved the P absorption and root P accumulation, establishing a stable percentage of stem P accumulation, the total biomass of leaves and seedlings, seed cotton yield and fiber length [65,66]. However, in another study, it was found that the low-P-tolerant variety showed higher fiber strength, fiber fineness, fiber uniformity, and fiber elongation, although it was found that different P concentrations had no direct impact on fiber quality [67]. Several reports have shown that, compared with the low-P sensitive cultivar, the low-P-tolerant cultivar had high cotton-seed weight, cotton-seed yield, and dry biomass, and accumulated more P [68,69,70,71]. Other studies have shown that low-phosphorus conditions will also have a greater impact on the content of large amounts, and of the micronutrients in cottonseed [72].

## 5. Conclusions

Low P availability reduced leaf area, shoot length, and dry weight, particularly in low-P-sensitive genotypes. Similarly, low P decreased pigment content and net photosynthetic rate, and P-use efficiency of cotton genotypes, especially low-P-sensitive genotypes. The activity of the PSII, ATP, and NADP(H) content decreased under low-P conditions. Moreover, the analysis of ChlF parameters showed that low P availability impairs the electron transport from the donor side of the PSII to the receptor side of the PSI terminal, which has a greater impact on the performance of the PSII donor side than the PSII and the PSI receptor side. Low P availability increased the content of malondialdehyde in leaves but decreased the activities of superoxide dismutase and catalase, especially in low-sensitive genotypes. Based on these results, we concluded that low P availability affects nutrient absorption, reduces photosynthetic performance, and leads to ROS production. Different low-P-tolerant cotton genotypes showed differences in their adaptability to low P availability. This may be the key reason for the difference in low-P tolerance among these cotton genotypes. Moreover, this research confirms that under stress conditions, the fall in the I-step of the induction curve for the early diagnosis of P deficit in cotton is a unique physiological bioindicator. Our findings may help to better understand the theoretical basis of the effect of low P availability on the photosynthesis and antioxidant system in cotton.

## Data Availability

Data are contained within the article and Supplementary Materials.

## Supplementary Materials

The following supporting information can be downloaded at https://www.mdpi.com/article/10.3390/antiox12020466/s1, Figure S1: Changes in (A) P accumulation, (B) leaf area in cotton leaves under different P treatments; Figure S2: Changes in (A) chlorophyll a, (B) chlorophyll b, (C) total chlorophyll, (D) chlorophyll a/b ratio, (E), carotenoid and (F) total chlorophyll/carotenoid ratio in cotton leaves under different P treatments; Figure S3: Effects of different P treatments on O-J-I-P phase among cotton genotypes; Figure S4: Pipeline models showing relative changes in energy flows per reaction center (left panel) and per active-leaf cross section (right panel) after 15, 50, and 500 μM P content treatment of a cotton seedling leaf. Table S1: Formulae and explanation of the technical data of the OJIP curves and the selected JIP-test parameters used in this study; Table S2: Growth parameters, PUE parameters, and PPUE under different phosphorus treatments; Table S3: Antioxidants, PEPC-enzyme activities, and MDA, ATP, NADP(H) content under different phosphorus treatments; Table S4: Gas-exchange parameters and contents of chlorophyll and carotene under different phosphorus treatments; Table S5: Chlorophyll-fluorescence parameters under different phosphorus treatments. Table S6: Global ANOVA of various characteristics in different phosphorus conditions.
