# Supplementary material for: Phosphorus Availability Affects the Photosynthesis and Antioxidant System of Contrasting Low-P-Tolerant Cotton Genotypes
Source: Antioxidants (Basel). 2023 Feb 12;12(2):466. doi: 10.3390/antiox12020466 (PMC9952849; doi:10.3390/antiox12020466)
Supplement: Supplementary file 1 [file antioxidants-12-00466-s001.zip › antioxidants-2028947-Supplemental figures.pdf]

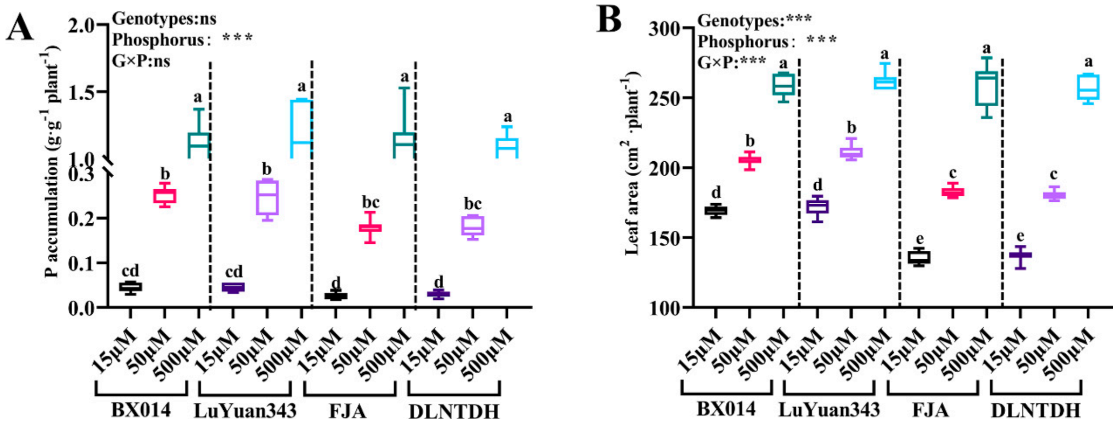

Figure S1. Changes in (A) P accumulation, (B) leaf area in cotton leaves under different P treatments. The results are expressed as means± SE (n=7). Statistically significant changes are indicated by different letters using the two-way ANOVA and multiple comparison test (P ≤0.05). P-values of the ANOVAs of genotypes (G), phosphorus level (P), and their interaction (G×P) are indicated as ns, not significant; ***: $P < 0.001$.

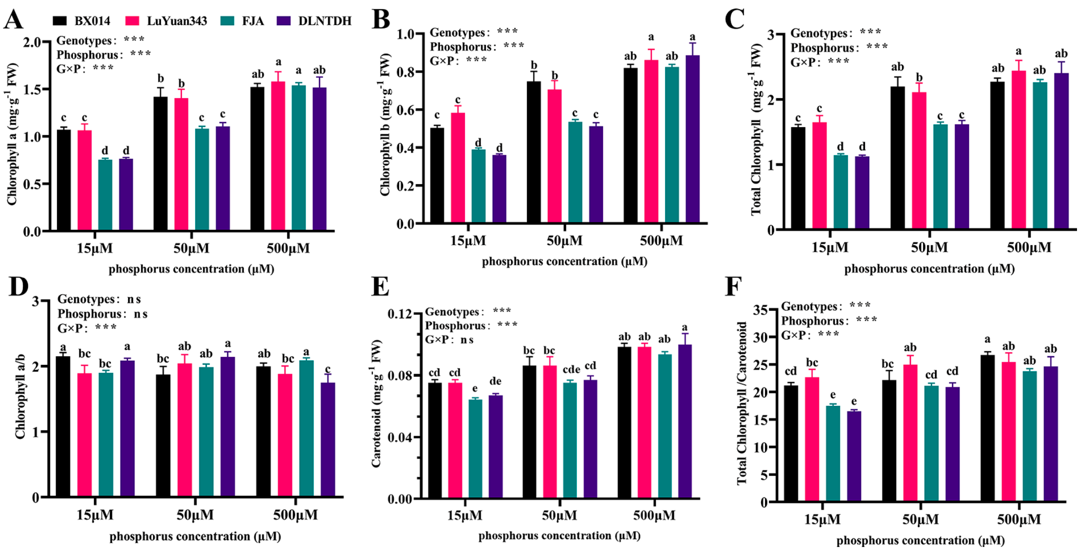

Figure S2. Changes in (A) chlorophyll a, (B) chlorophyll b, (C) total chlorophyll, (D) chlorophyll a/b ratio, (E), carotenoid and (F) Total chlorophyll/carotenoid ratio in cotton leaves under different P treatments. The results are expressed as means± SE (n=7). Statistically significant changes are indicated by different letters using the two-way ANOVA and multiple comparison test (P ≤0.05). P-values of the ANOVAs of genotypes (G), phosphorus level (P), and their interaction (G×P) are indicated as ns, not significant; ***: $P < 0.001$.

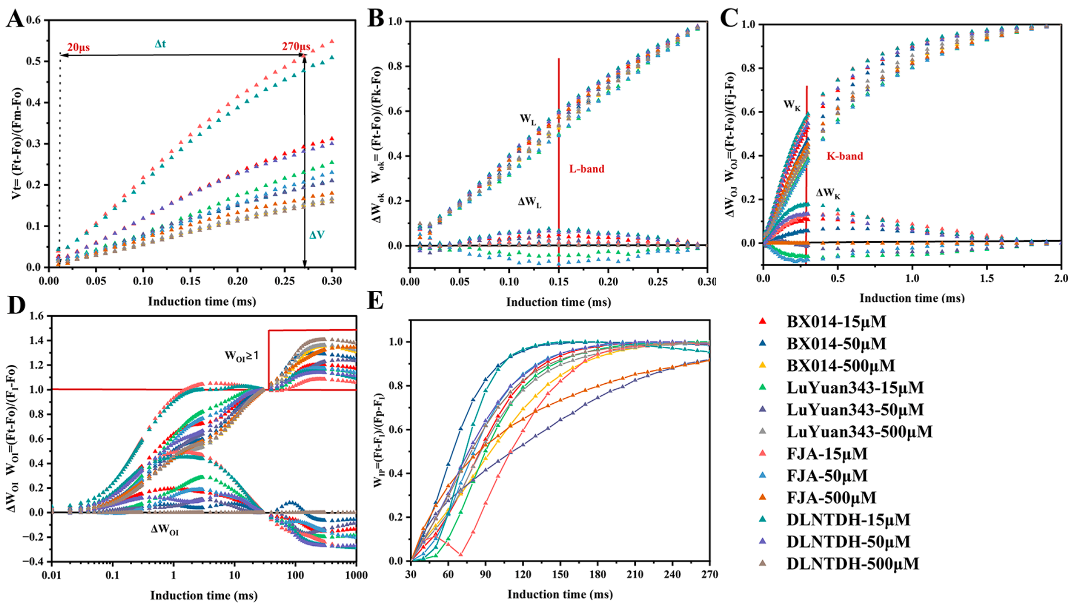

Figure S3. Effects of different P treatments on O-J-I-P phase among cotton genotypes. (A) Fluorescence relative variation Vt vs. Time, from 20 μs to 300 μs in a linear time scale to show the initial slope. (B) The fluorescence rise kinetics normalized by Fo and F_K as W_{OK} and the difference kinetics ΔW_{OK} in a linear time scale from 0 to 0.30 ms. (C) The fluorescence rise kinetics normalized by Fo and F_J as W_{OJ}, and the difference kinetics ΔW_{OJ} in a linear time scale from 0 to 2 ms. (D) The fluorescence rise kinetics curves normalized by Fo and F_I as W_{OI} = (Ft − Fo)/(F_I − Fo) and the difference kinetics ΔW_{OI} in a logarithmic time scale. (E) Fluorescence increase kinetics curves with FI and FP normalized as a weighted average.

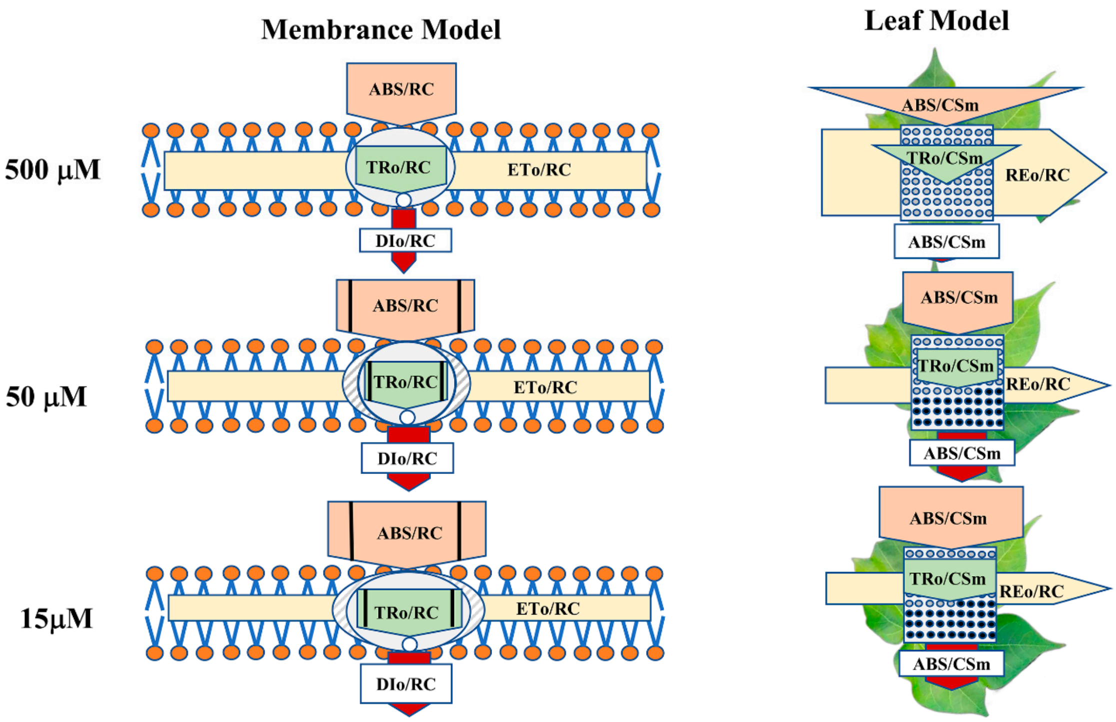

Figure S4. Pipeline models showing relative changes in energy flows per reaction center (left panel) and per active leaf cross section (right panel) after 15, 50, and 500 μM P content treatment of a cotton seedling leaf. The relative change in the width of each arrow shows how each parameter responded. Active RCS are shown as open circles, whereas inactive RCS are displayed as filled black circles.
